# Effect of blood pressure on mortality in patients with cognitive impairment: a prospective cohort study

**DOI:** 10.3389/fcvm.2023.1282131

**Published:** 2023-12-12

**Authors:** YanChang Shang, ShuHui Wang, Chao Wei, ZhongBao Gao, HengGe Xie, ZhenFu Wang

**Affiliations:** ^1^ Department of Geriatric Neurology, The Second Medical Center & National Clinical Research Center for Geriatric Diseases, Chinese PLA General Hospital, Beijing, China; ^2^ Department of Neurology, Beijing Friendship Hospital, Capital Medical University, Beijing, China

**Keywords:** cognitive impairment, hypertensive, mortality, prospective study, NHANES (National Health and Nutrition Examination Survey)

## Abstract

**Background:**

Cognitive impairment is a prevalent condition that substantially elevates mortality rates among the elderly. The impact of hypertension on mortality in older adults with cognitive impairment is a subject of contention. This study aims to examine the influence of hypertension on both all-cause and CVD-specific mortality in elderly individuals experiencing cognitive impairment within a prospective cohort.

**Methods:**

This study encompassed 2,925 participants (weighted 53,086,905) aged 60 years or older from National Health and Nutrition Examination Survey (NHANES) spanning 2011–2014. Incidence of all-cause and CVD-specific mortality was ascertained through linkage with National Death Index records until 31 December 2019. Survival was performed employing the Kaplan–Meier method. Hazard ratios (HRs) were calculated via Cox proportional hazards regression models.

**Results:**

Over the follow-up period of up to 9.17 years [with a median (IQR) time to death of 6.58 years], equivalent to 18,731.56 (weighted 3.46 × 10^8^) person-years, there were a total of 576 recorded deaths. Participants with CI exhibited a 1.96-fold higher risk of all-cause mortality (95% CI: 1.55–2.49; *p* < 0.01) and a 2.8-fold higher risk of CVD-specific mortality (95% CI: 1.83–4.29; *p* < 0.01) in comparison to participants without CI. Among participants with CI, concurrent hypertension comorbidity was linked to a 2.73-fold elevated risk of all-cause mortality (95% CI: 1.78–4.17; *p* < 0.01) and a 5.3-fold elevated risk of CVD-specific mortality (95% CI: 2.54–11.04; *p* < 0.01). Further stratified analyses revealed that the combined effects of hypertension and CI on all-cause and CVD-specific mortality were more pronounced in participants aged 60–69 years compared to those aged 70–80 years (*p* for interaction <0.01). The primary findings exhibited resilience across a series of sensitivity analyses.

**Conclusions:**

Participants with CI exhibited a markedly elevated risk of all-cause and CVD-specific mortality when coexisting with hypertension. Appropriate management of hypertension in patients with CI may be helpful in reducing the excess risk of death.

## Introduction

Cognitive impairment (CI), encompassing Alzheimer's disease and other forms of dementia, constitutes a substantial and escalating global health concern and burden, given the accelerated pace of global aging and increased life expectancies. As highlighted in a 2018 report by the World Health Organization, approximately 50 million individuals worldwide were afflicted by dementia, with an annual global incidence of approximately 9.9 million new cases (1, 2). Projections indicate that these figures will soar to 82 million by 2030 and 152 million by 2050 (3). Moreover, the economic costs associated with dementia on a global scale were estimated to have reached an astounding US $1,313.4 billion in 2019, exerting immense pressure on both care systems and families alike (4).

Several studies have consistently demonstrated a higher mortality rate in patients with CI compared to the general population with normal cognitive function. A meta-analysis, encompassing 52,134 individuals, revealed a pooled multivariate-adjusted risk ratio (RR) of 1.48 (95% CI: 1.36–1.61) for all-cause mortality in women and 1.34 (95% CI: 1.24–1.44) in men, when comparing those with CI to those without CI (5). The Health and Retirement Study revealed that non-Hispanic Black participants experienced a significantly higher mortality burden from dementia (24.7%; 95% CI: 17.3–31.4) compared to non-Hispanic White participants (12.2%; 95% CI: 10.7–13.6) (6). Moreover, CI was associated with an increased risk of death within one year for patients undergoing abdominal aortic aneurysm repair, coronary artery bypass grafting, or colectomy (7).

Many individuals who have CI tend to present concurrently with other conditions such as hypertension, diabetes, and metabolic syndrome, particularly among the frail older population (8, 9). Hypertension and diabetes are acknowledged as independent risk factors for CI. Several studies suggest that diseases such as hypertension and diabetes might disrupt endothelial cell function and contribute to the development of cognitive dysfunction (10–12). Furthermore, the coexistence of hypertension and CI may heighten the mortality risk among patients. However, the findings from studies investigating the impact of blood pressure on mortality in individuals with CI yielded inconsistent results (13, 14). Hence, the objective of this current study is to investigate the association between blood pressure levels and mortality rates among individuals with CI, utilizing data collected from a nationally representative population survey.

## Material and methods

### Study participants

**Figure 1:**
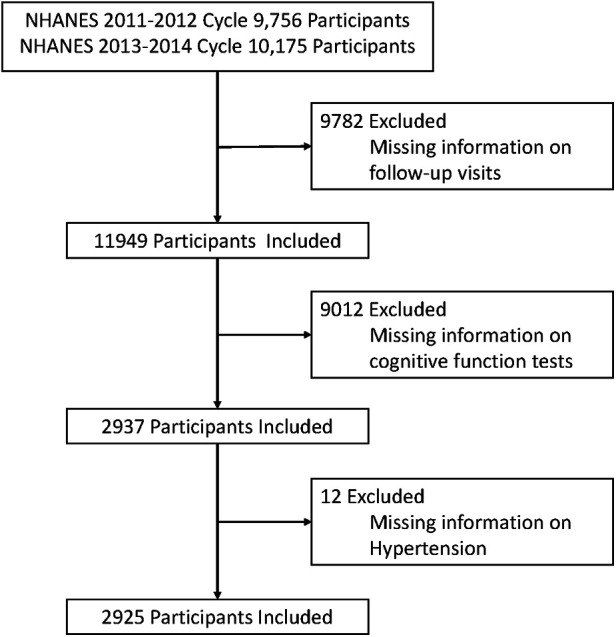
Flow chart for selecting study participants from the 2011 to 2014 cycles of the NHANES.

The National Center for Health Statistics (NCHS) of the Centers for Disease Control and Prevention (CDC) conducts the NHANES, a comprehensive health survey of the civilian, non-institutionalized population in the United States (15). Each survey respondent undergoes a wphysical examination in a mobile examination center and a residential interview as part of a large-scale, multistage, ongoing, and nationally representative methodology. The NHANES has been authorized by the NCHS Ethics Review Board, and all participants have provided written informed consent. This investigation followed the STROBE (Strengthen the Reporting of Observational Studies in Epidemiological Studies) declaration (16). For this cohort analysis, we enrolled 19,931 participants from two NHANES cycles conducted between 2011 and 2014. We excluded 9,782 participants with missing follow-up information, 9,012 participants with missing cognitive function test information, and 12 participants with missing hypertension information from this study. Finally, a total of 2,925 participants were included in this study (see Figure 1).

### Determination of mortality

The mortality data utilized in this investigation to assess fatalities were sourced from the linked mortality files (LMF), which are made accessible to the public through the NCHS Research Data Center (RDC) (17). The public-use version of the LMF included data on fatalities that occurred between the time of survey participation and 31 December 2019. For this study, all cause of death, regardless of the underlying cause, were considered under the criteria of all-cause mortality. To describe cardiovascular disease (CVD) mortality, the study employed codes I00–I09, I11, I13, I20–I51, and I60–I69 from the International Statistical Classification of Diseases and Related Health Problems, Tenth Revision. This approach ensured a consistent identification of CVD fatalities within the dataset.

### Evaluation of cognitive performance and identification of CI

The NHANES employed three distinct measures to assess cognitive function. The initial test, known as the Consortium to Establish a Registry for Alzheimer's disease Word Learning (CERAD-WL) subtest, focused on delayed memory. Participants were instructed to recall as many of the randomly selected set of 10 words as possible after undergoing three consecutive learning trials. The second evaluation, called the Animal Fluency (AF) exam, gauged verbal fluency. In this assessment, participants were given one minute to generate a list of as many animals as they could. The third test was the Digit Symbol Substitution Test (DSST), which measured processing speed and sustained attention. Participants completed this test on a paper form, where they had 120 s to replicate symbols from a provided key. After completing the AF and DSST examinations, participants were administered the CERAD-Delayed Recall Test (CERAD-DR). This test assessed the participants' memory of the 10 unrelated words from the initial round of trials. Scores for this test were measured on a scale of 0–10 points. Participants were given practice tests prior to the exams. For the verbal fluency test, participants were specifically instructed to name three items of clothing. If participants were unable to comply with this instruction, they were excluded from proceeding to the AF test. Similarly, those who were unable to match symbols with numbers during the pretest practice were also disqualified from further participation. To provide an overall measure of cognitive ability, each of the individual test results were standardized using Z score transformations. These standardized scores from the three tests were summed to calculate the composite Z score. If a subject's composite score fell more than 1 standard deviation below the mean, they were classified as having CI (18).

### Sociodemographic characteristics, lifestyle factors, and comorbidities

Household interviews were conducted to collect data on potential confounding variables, such as comorbidities, lifestyle factors, and sociodemographic characteristics. Sociodemographic factors included age, gender (male or female), race (Mexican American, Other Hispanic, Non-Hispanic White, Non-Hispanic Black, Other Race), educational attainment, and family income. Educational level was categorized into three tiers on the degree of education: <high school level, high school or equivalent level, and some college or more level. Family income was classified into three tiers based on the family poverty income ratio (<1.0, 1.0–3.0, or >3.0). Alcohol use, smoking, and physical exercise were all lifestyle factors. Participants who claimed to have smoked at least 100 cigarettes in their lives were considered smokers. Alcohol drinkers were those who consumed at least 12 alcoholic beverages annually. Engaging in moderate/vigorous-intensity exercise that resulted in significant increases in respiration or heart rate for at least 10 min continuously during a typical week was deemed to be moderate/vigorous work activity. The self-reported history, self-reported antihypertensive drug usage, or uncontrolled blood pressure (>140/90 mm Hg) on physical examination were used to identify hypertension. The self-reported history of coronary artery disease or myocardial infarction was used to diagnose coronary artery disease. The self-reported history or antidiabetic medication usage was used as criteria to define diabetes. The self-reported history or usage of cholesterol-modifying medications was used as a basis to define hyperlipidemia. The self-reported history of stroke and any type of tumors were utilized to define stroke and tumors.

### Statistical analysis

Due to the complex sampling design of the NHANES, all analyses in the current study took sample weights, clustering, and stratification into account in accordance with the NHANES statistical analysis guidelines (15). The Kolmogorov–Smirnov test was utilized to assess the normal distribution of continuous variables. Using *t*-tests, *χ*^2^ tests, or Mann–Whitney *U*-tests, as appropriate, we compared the differences in continuous variables or categorical variables between groups. Survival analysis was conducted using the Kaplan–Meier technique, and any differences in survival were evaluated through a stratified log-rank test. Subsequently, multivariable Cox proportional hazards regression models were employed to calculate hazard ratios (HRs) and 95% confidence intervals (CIs) to examine the associations of hypertension and CI with risks of all-cause and CVD mortality. The assumption of proportional hazards was assessed using Schoenfeld residuals and no violations were detected. Person-years were computed for each participant from the study commencement until death or the end of the follow-up period (31 December 2019). Three multivariable models were constructed. Model 1 was adjusted for age (<70 years, ≥70 years), gender (male, female), race (Mexican American, Other Hispanic, Non-Hispanic White, Non-Hispanic Black, Other Race), educational attainment (< high school, high school or equivalent, or college), and the family poverty income ratio value (<1.0, 1.0–3.0, or >3.0). Model 2 further incorporated adjustments for smoking, alcohol use, moderate physical activity, and intense physical activity. Model 3 additionally included adjustments for the history of diabetes, hypercholesterolemia, cardiovascular disease, stroke, and malignancies. The construction of these models for regression analysis was based on a combination of literature review, expert consultations, and preliminary analyses. Missing values were imputed using multiple imputation. Subgroup analyses were performed stratified by age, gender, education level, moderate physical activity, and vigorous physical activity to explore potential effect modifications. Interaction terms between hypertension and CI, as well as stratification variables, were assessed through the *p*-values for the interaction terms. Moreover, the interaction between hypertension and impaired cognition was evaluated.

Multiple sensitivity analyses were conducted. Using the original, non-imputed data, the principal analysis was initially performed. To validate the main findings, additional participants with a history of diabetes, hypercholesterolemia, stroke, or malignancies were excluded.

For two-sided testing, a statistical significance threshold of *p* < 0.05 was employed. Data analysis was conducted using SPSS (version 23.0, IBM Corp.) and R (version 4.2.0) between November 2022 and April 2023.

## Results

The present study included 2,925 individuals, representing a weighted sample of 53,086,905 Americans aged 60 years and older [weighted mean (SD) age, 69.2 (6.6) years; 45.5% male, see Table 1]. There were 1,397 non-Hispanic Whites (weighted percentage: 79.5%), 698 non-Hispanic Blacks (weighted percentage: 8.4%), 256 Mexican Americans (weighted percentage: 3.4%), 293 Hispanics (weighted percentage: 3.6%), and 281 other races or ethnicities (weighted percentage: 5.0%). The prevalence of hypertension among this older group was approximately 59.1% (weighted percentage). In comparison to those without hypertension, individuals with hypertension were significantly older, with a higher percentage of individuals aged 70–80, and a higher percentage of women (*p* < 0.01). Participants with hypertension were also more likely to be non-Hispanic Black, have lower education levels (below high school level), and have a family income-to-poverty ratio below 3.0 compared to participants without hypertension (*p* < 0.01). Regarding lifestyle parameters, the proportion of smokers and Vigorous physical activity were significantly lower in the hypertensive population than in the non-hypertensive population (*p* < 0.05), while the proportion of alcohol drinkers and moderate physical activity did not differ significantly between the two groups (*p* > 0.05). In terms of co-morbidities, the frequency of diabetes mellitus, CVD, stroke, and tumors was significantly higher in the hypertensive population than in the non-hypertensive population (*p* < 0.01), while the prevalence of hyperlipidemia was significantly lower. Notably, compared to the non-hypertensive group, the proportion of CI was nearly twice as high in the hypertensive population (weighed percentage: 12.8% vs. 6.8%, *p* < 0.01).

**Table 1:** Baseline characteristics of participants with and without hypertension, NHANES, 2011–2014.

Characteristic	Total *N* = 2,925 (weighted%)	No hypertension *N* = 1,096 (weighted%)	Hypertension *N* = 1,829 (weighted%)	*p*-value
Age, years				<0.001
mean (SD)	69.20 (6.66)	68.14 (6.33)	69.94 (6.78)	
60–69	1,586 (56.0)	658 (63.3)	928 (51.0)	
70–80	1,339 (44.0)	438 (36.7)	901 (49.0)	
Sex				<0.001
Male	1,422 (45.5)	581 (48.8)	841 (43.3)	
Female	1,503 (54.5)	515 (51.2)	988 (56.7)	
Race				<0.001
Mexican American	256 (3.4)	111 (3.5)	145 (3.3)	
Other hispanic	293 (3.6)	132 (3.9)	161 (3.4)	
Non-hispanic white	1,397 (79.5)	541 (82.1)	856 (77.7)	
Non-hispanic black	698 (8.4)	189 (5.3)	509 (10.6)	
Other race	281 (5.0)	123 (5.2)	158 (4.9)	
Education status				<0.001
<High school	744 (16.0)	251 (13.1)	493 (18.0)	
High school	1,506 (53.5)	532 (50.2)	974 (55.7)	
Some college or above	675 (30.5)	313 (36.7)	362 (26.3)	
Family income to poverty ratio				0.003
<1.0	515 (9.6)	173 (8.3)	342 (10.5)	
1.0–3.0	1,301 (39.5)	465 (36.2)	836 (41.8)	
>3.0	1,109 (50.9)	458 (55.5)	651 (47.7)	
Smoking status				0.083
Yes	372 (11.0)	155 (12.5)	217 (9.9)	
No	2,553 (89.0)	941 (87.5)	1,612 (90.1)	
Alcohol drinker				0.018
Yes	1,987 (72.5)	774 (74.6)	1,213 (71.0)	
No	938 (27.5)	322 (25.4)	616 (29.0)	
Vigorous physical activity				0.017
Yes	308 (12.7)	135 (13.8)	173 (11.9)	
No	2,617 (87.3)	961 (86.2)	1,656 (88.1)	
Moderate physical activity				0.368
Yes	798 (31.6)	310 (33.3)	488 (30.4)	
No	2,127 (68.4)	786 (66.7)	1,341 (69.6)	
Diabetes				<0.001
Yes	963 (26.9)	252 (17.2)	711 (33.6)	
No	1,962 (73.1)	844 (82.8)	1,118 (66.4)	
Hypercholesterolemia				<0.001
Yes	1,285 (42.2)	619 (54.3)	666 (33.9)	
No	1,640 (57.8)	477 (45.7)	1,163 (66.1)	
Coronary heart disease				<0.001
Yes	266 (9.4)	49 (4.9)	217 (12.5)	
No	2,659 (90.6)	1,047 (95.1)	1,612 (87.5)	
Stroke				<0.001
Yes	202 (6.4)	37 (3.3)	165 (8.6)	
No	2,723 (93.6)	1,059 (96.7)	1,664 (91.4)	
Tumor				0.005
Yes	595 (24.1)	193 (20.8)	402 (26.4)	
No	2,330 (75.9)	903 (79.2)	1,427 (73.6)	
Cognitive impairment				0.001
Yes	479 (10.3)	146 (6.8)	333 (12.8)	
No	2,446 (89.7)	950 (93.2)	1,496 (87.2)	

### Association hypertension and CI with all-cause and CVD-specific mortality

**Figure 2:**
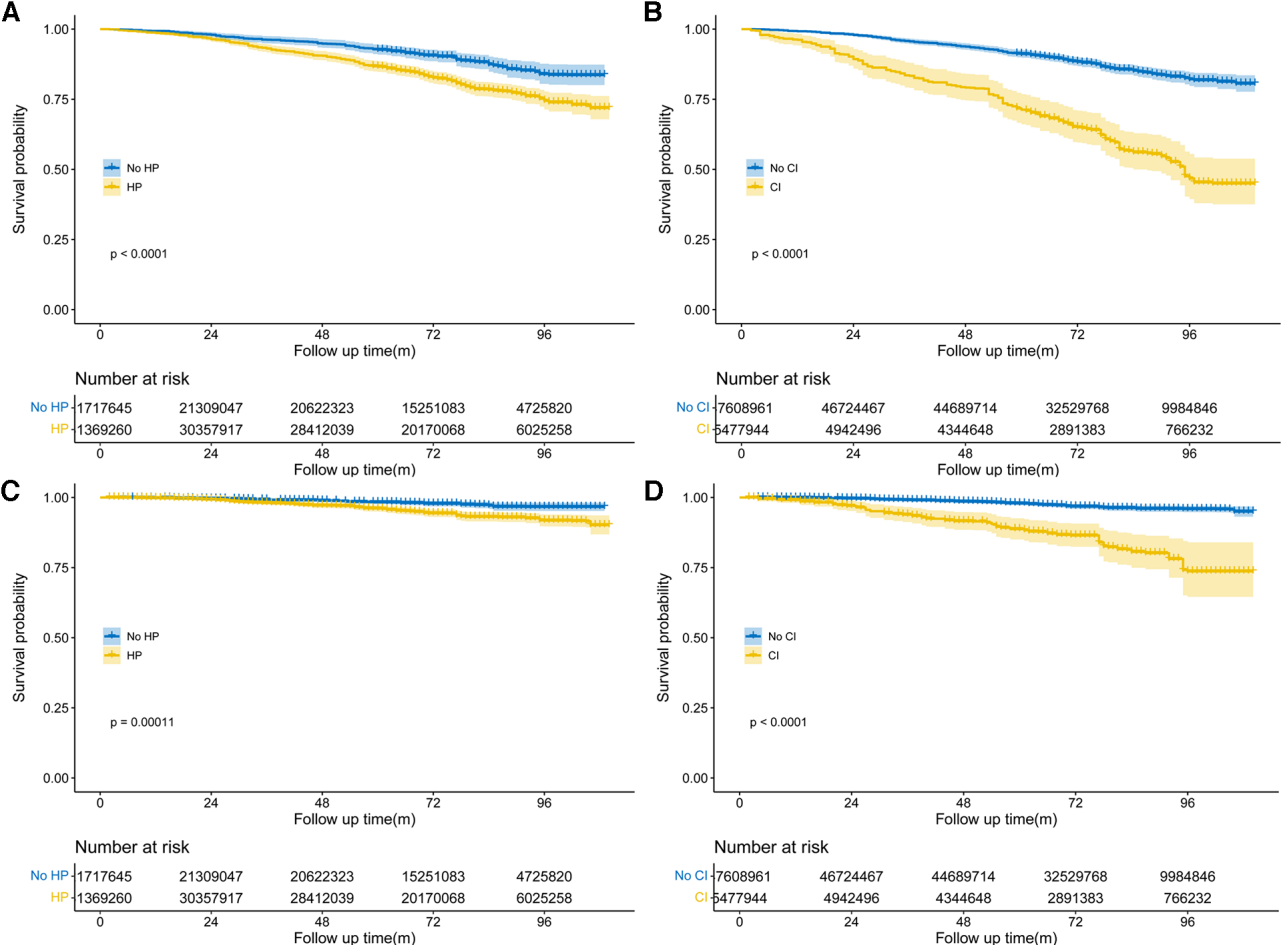
Weighted Kaplan–Meier curves for all-cause and CVD-specific mortality in different groups, stratified by hypertension (**A,C**), cognitive impairment status (**B,D**). *P*-value calculated from log-rank statistic.

**Figure 3:**
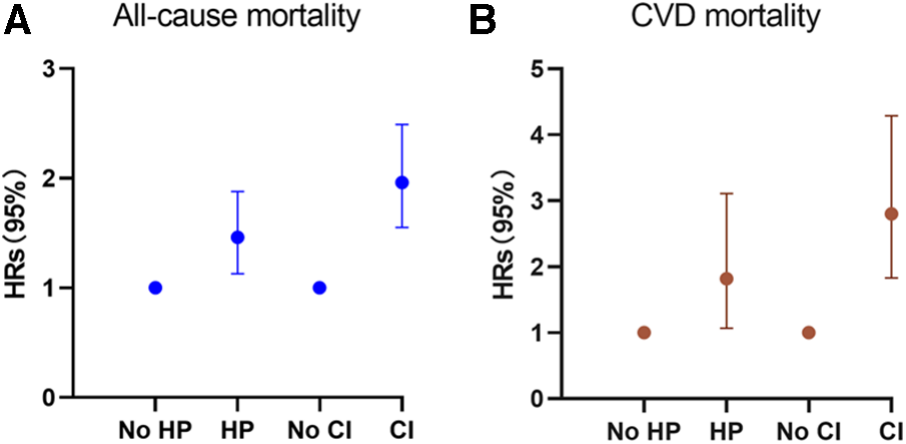
Hazard ratios (solid symbols) with 95% CIs (error bars) of hypertension and cognitive impairment with all-cause (**A**) and CVD-specific (**B**) mortality were estimated using weighted multivariable cox regression models adjusted for sociodemographic characteristics, lifestyle factors, and comorbidities among participants, NHANES, 2011–2014.

During the follow-up period of up to 9.17 years [median (IQR) time to death: 6.58 years], or 18,731.56 [weighted 3.46 × 10^8^] person-years, a total of 576 deaths occurred. Among them, 156 (27.08%) individuals perished from CVD, 149 (25.87%) participants perished from cancers, and 271 (47.05%) participants perished from various other reasons. All-cause mortality was significantly higher in individuals with hypertension compared to those without hypertension [3.65 (95% CI: 1.85–2.53) events per 100 patient-years vs. 2.16 (95% CI: 3.31–4.01) events per 100 patient-years; *p* < 0.01; see Figure 2A]. Stratified log-rank test demonstrated that individuals with hypertension also had significantly higher CVD-specific mortality than those without it [1.03 (95% CI: 0.86–1.24) vs. 0.51 (95% CI: 0.37–0.71) events per 100 patient-years; p < 0.01; see Figure 2C]. After adjusting for age, sex, and race (Model 1), Cox regression analysis revealed that individuals with hypertension had a 1.52-fold (95% CI: 1.17–1.98; *p* < 0.01) higher risk of all-cause death than participants without hypertension (see Table 2). Participants with hypertension exhibited a 1.51-fold (95% CI: 1.17–1.96; *p* < 0.01) and 1.46-fold (95% CI: 1.13–1.88; *p* < 0.01) greater risk of all-cause death, respectively, after further adjustment for lifestyle variables (Model 2) and comorbidities (Model 3, see Figure 3A). Comparatively, individuals with hypertension had a significantly higher risk of CVD-specific death compared to those without hypertension, with hazard ratios of 2.14 (*p* < 0.01), 2.08 (*p* < 0.01), and 1.82 (*p* < 0.01).

**Table 2:** Hazard ratios of hypertension and cognitive impairment with all-cause and CVD-specific mortality among participants, NHANES, 2011–2014.

	HR (95% CI)		HR (95% CI)	
	No hypertension	Hypertension	No cognitive impairment	Cognitive impairment
All-cause mortality				
Deaths, No./Total No.	156/1,096	420/1,829	397/2,446	179/479
Model 1	1 (Reference)	1.52 (1.17–1.98)	1 (Reference)	2.5 (1.96–3.19)
Model 2	1 (Reference)	1.51 (1.17–1.96)	1 (Reference)	2.04 (1.63–2.56)
Model 3	1 (Reference)	1.46 (1.13–1.88)	1 (Reference)	1.96 (1.55–2.49)
CVD mortality				
Deaths, No./Total No.	37/1,096	119/1,829	96/2,446	60/479
Model 1	1 (Reference)	2.14 (1.27–3.59)	1 (Reference)	3.85 (2.69–5.49)
Model 2	1 (Reference)	2.08 (1.22–3.52)	1 (Reference)	2.92 (1.91–4.46)
Model 3	1 (Reference)	1.82 (1.07–3.11)	1 (Reference)	2.8 (1.83–4.29)

Model 1: Adjusted for age, sex, race-ethnicity, and education levels; Model 2: Adjusted for variables in Model 1 plus smoking status, drinking status, vigorous, and moderate physical activity; Model 3: Adjusted for variables in Model 2 plus a history of diabetes, hypercholesterolemia, cardiovascular disease, stroke, and tumors.

We also examined the impact of CI on participant mortality, including all-cause death and mortality specifically related to CVD. The findings of the stratified log-rank test revealed that patients with CI had a significantly worse overall survival rate compared to people without CI [6.38 (95% CI: 5.51–7.39) events per 100 patient-years vs. 2.49 (95% CI: 2.26–2.75) events per 100 patient-years; *p* < 0.01; see Figure 2B]. Additionally, those with CI also demonstrated substantially higher rates of CVD-specific mortality compared to individuals without CI [2.14 (95% CI: 1.66–2.75) events per 100 patient-years vs. 0.60 (95% CI: 0.49–0.74) events per 100 patient-years; *p* < 0.01; see Figure 2D]. Further Cox regression analysis, controlling for sociodemographic characteristics (Model 1), revealed that participants with CI had a 2.50-fold higher risk of all-cause mortality compared to those without CI (see Table 2). Moreover, participants with CI had a significantly elevated risk of all-cause mortality that was 2.04-fold (95% CI: 1.63–2.56; *p* < 0.01) and 1.96-fold (95% CI: 1.55–2.49; *p* < 0.01) after adjustment for lifestyle variables (Model 2) and comorbidities (Model 3, see Figure 3B), respectively. When comparing individuals with CI to those without CI, there was a 3.85-fold, 2.92-fold, and 2.8-fold higher risk of CVD-specific mortality.

### Joint analysis of hypertension and CI with all-cause and CVD-specific mortality

**Figure 4:**
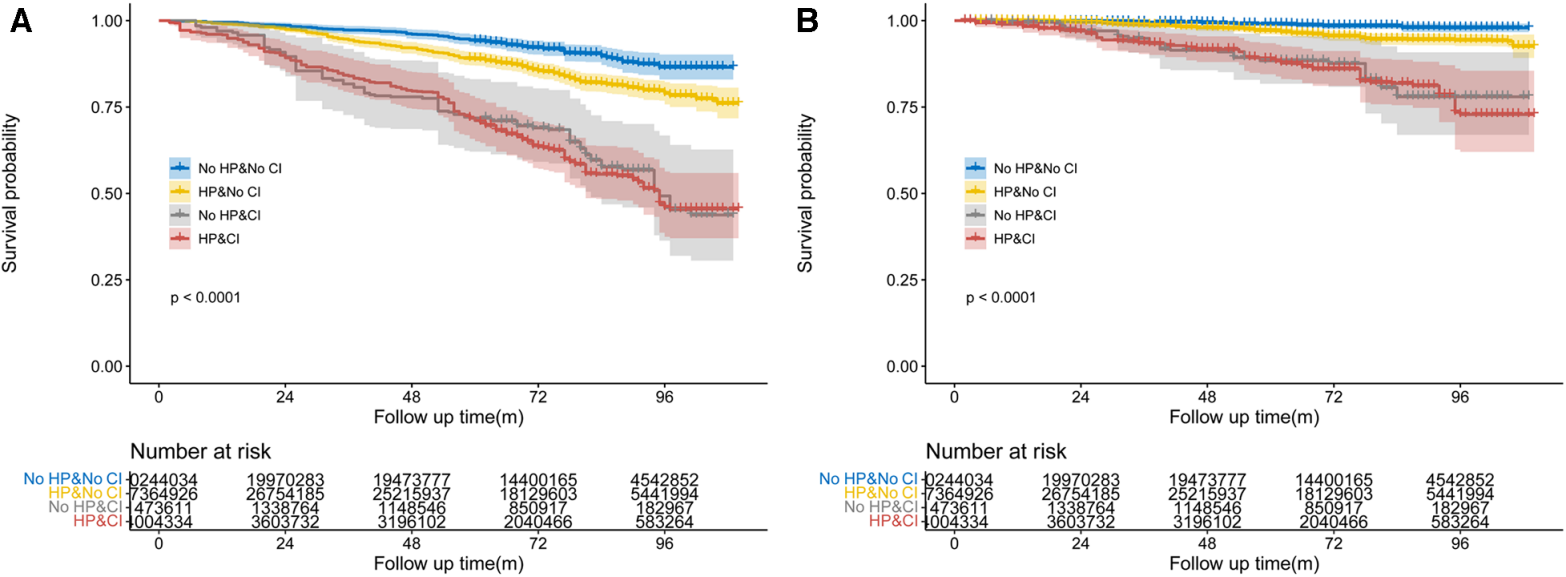
Weighted Kaplan–Meier curves for All-cause (**A**) and CVD-specific (**B**) mortality in different groups, stratified by the combination of diabetes and cognitive impairment. *P*-value calculated from log-rank statistic.

**Figure 5:**
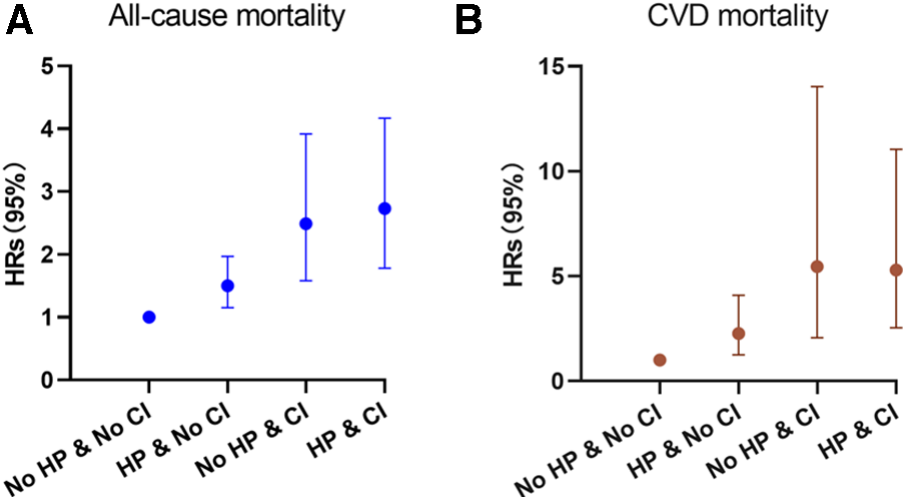
Hazard ratios (solid symbols) with 95% CIs (error bars) of hypertension combined with cognitive impairment on all-cause (**A**) and CVD-specific (**B**) mortality were estimated using weighted multivariable cox regression models adjusted for sociodemographic characteristics, lifestyle factors, and comorbidities among participants, NHANES, 2011–2014.

We investigated the potential impact of both CI and hypertension on mortality from all causes and specifically CVD. The study categorized individuals into four groups based on the presence of CI and hypertension, with individuals without either condition serving as the reference group. Analysis using the stratified log-rank test demonstrated significant differences in both all-cause mortality and CVD-specific mortality among the four groups (*p* < 0.01; see Figure 4A). Among participants, those without both hypertension and CI exhibited the lowest risk of overall death, with an incidence rate of 1.55 (95% CI: 1.23–1.99) events per 100 patient-years. This was followed by individuals with hypertension alone, who had an incidence rate of 2.74 (95% CI: 2.37–3.18) events per 100 patient-years. Those with CI only had a substantially higher incidence rate of 7.35 (95% CI: 5.34–10.37) events per 100 patient-years. Notably, the group with both hypertension and CI faced the highest risk of mortality, with an incidence rate of 7.94 (95% CI: 6.57–9.65) events per 100 patient-years (*p* < 0.01). Similarly, individuals with both CI and hypertension had the highest risk of CVD-specific mortality with an incidence rate of 2.93 (95% CI: 2.10–4.20) events per 100 patient-years (*p* < 0.01, see Figure 4B) across all four categories. To assess the relationship between illness conditions and the risk of mortality from all causes and specifically CVD, three Cox proportional hazards models were constructed. After adjusting for sociodemographic characteristics, the analysis revealed that individuals with hypertension or CI alone faced a higher risk of all-cause mortality [1.55-fold (95% CI: 1.16–2.07; *p* < 0.01) and 3.13-fold (95% CI: 2.0–4.91; *p* < 0.01), respectively; see Table 3]. For those with both diseases, the risk increased even further (3.44-fold; 95% CI: 2.23–5.3; *p* < 0.01). Additionally adjusting for lifestyle factors and comorbidities demonstrated that participants with both conditions had a 2.73-fold higher risk of all-cause mortality compared to participants without either condition (95% CI: 1.78–4.17; *p* < 0.01; see Figure 5A), which was also significantly higher than the risk observed among those with hypertension or CI alone. The fully adjusted Cox modeling analysis showed that individuals with both CI and hypertension had the highest risk of CVD-specific death, with a 5.3-fold increase (95% CI: 2.54–11.04) in comparison to the other group (see Table 3 and see Figure 5B).

**Table 3:** Hazard ratios of hypertension combined with cognitive impairment on all-cause and CVD-specific mortality among participants, NHANES, 2011–2014.

	HR (95% CI)		HR (95% CI)	
	No hypertension & No cognitive impairment	Hypertension & No cognitive impairment	No hypertension & Cognitive impairment	Hypertension & Cognitive impairment
All-cause mortality				
Deaths, No./Total No.	109/950	288/1,496	47/146	132/333
Model 1	1 (Reference)	1.55 (1.16–2.07)	3.13 (2.0–4.91)	3.44 (2.23–5.3)
Model 2	1 (Reference)	1.58 (1.19–2.09)	2.55 (1.63–3.98)	2.88 (1.87–4.44)
Model 3	1 (Reference)	1.5 (1.15–1.97)	2.49 (1.58–3.92)	2.73 (1.78–4.17)
CVD mortality				
Deaths, No./Total No.	20/950	76/1,496	17/146	43/333
Model 1	1 (Reference)	2.59 (1.46–4.6)	7.09 (2.76–18.25)	7.82 (3.96–15.44)
Model 2	1 (Reference)	2.61 (1.46–4.67)	5.49 (2.16–13.96)	6.05 (2.92–12.55)
Model 3	1 (Reference)	2.27 (1.26–4.1)	5.46 (2.07–14.43)	5.3 (2.54–11.04)

Model 1: Adjusted for age, sex, race-ethnicity, and education levels; Model 2: Adjusted for variables in Model 1 plus smoking status, drinking status, vigorous, and moderate physical activity; Model 3: Adjusted for variables in Model 2 plus a history of diabetes, hypercholesterolemia, cardiovascular disease, stroke, and tumors.

### Stratified and sensitivity analyses

**Figure 6:**
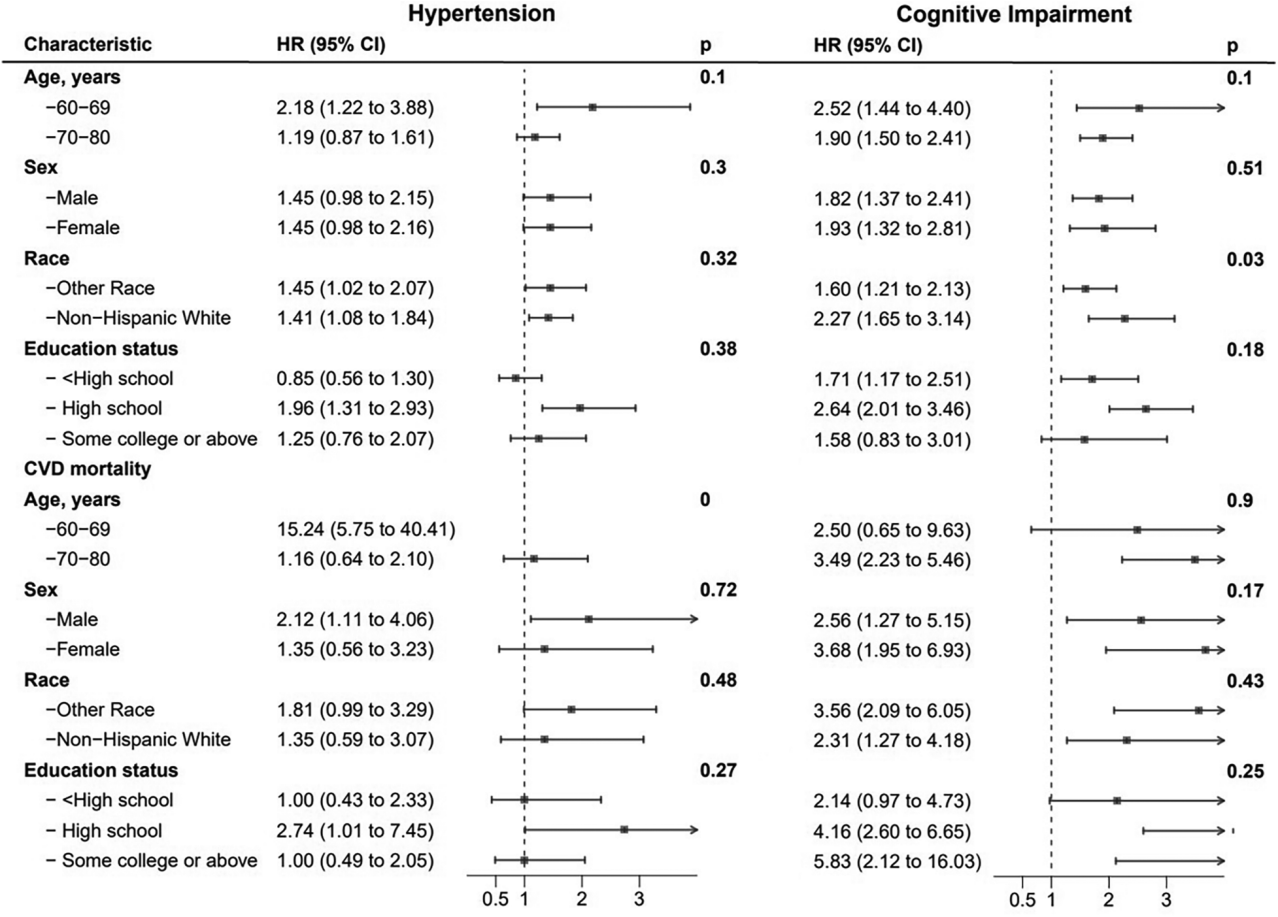
Hazard ratios of hypertension and cognitive impairment with all-cause and CVD-specific mortality in various subgroups among participants, NHANES, 2011–2014.

The results for different age, sex, race, and educational level groups were showed in Tables 4, 5. The joint associations of hypertension and CI with all-cause mortality were more pronounced in individuals aged 60–69 years, compared to those aged 70–80 years old (*p* for interaction <0.01, see Figure 6). Similarly, the associations between CVD-specific mortality and hypertension, either with or without comorbid CI, were stronger in younger age group compared to the older subpopulation (*p* for interaction <0.01, see Table 5). Further analyses that considered factors such as sex, race, and educational level did not reveal any significant interactions between hypertension and all-cause mortality, or between CI and all-cause mortality.

**Table 4:** Hazard ratios of hypertension and cognitive impairment with all-cause and CVD-specific mortality in various subgroups among participants, NHANES, 2011–2014.

Characteristic	Hypertension		Cognitive impairment	
	HR (95% CI)	*p* for interaction	HR (95% CI)	*p* for interaction
All-cause mortality				
Age, years		0.10		0.10
60–69	2.18 (1.22–3.88)		2.52 (1.44–4.4)	
70–80	1.19 (0.87–1.61)		1.9 (1.5–2.41)	
Sex		0.30		0.51
Male	1.45 (0.98–2.15)		1.82 (1.37–2.41)	
Female	1.45 (0.98–2.16)		1.93 (1.32–2.81)	
Race		0.32		0.03
Other race	1.45 (1.02–2.07)		1.6 (1.21–2.13)	
Non-hispanic white	1.41 (1.08–1.84)		2.27 (1.65–3.14)	
Education status		0.38		0.18
<High school	0.85 (0.56–1.3)		1.71 (1.17–2.51)	
High school	1.96 (1.31–2.93)		2.64 (2.01–3.46)	
Some college or above	1.25 (0.76–2.07)		1.58 (0.83–3.01)	
CVD mortality				
Age, years		0.00		0.90
60–69	15.24 (5.75–40.41)		2.5 (0.65–9.63)	
70–80	1.16 (0.64–2.1)		3.49 (2.23–5.46)	
Sex		0.72		0.17
Male	2.12 (1.11–4.06)		2.56 (1.27–5.15)	
Female	1.35 (0.56–3.23)		3.68 (1.95–6.93)	
Race		0.48		0.43
Other race	1.81 (0.99–3.29)		3.56 (2.09–6.05)	
Non-hispanic white	1.35 (0.59–3.07)		2.31 (1.27–4.18)	
Education status		0.27		0.25
<High school	1 (0.43–2.33)		2.14 (0.97–4.73)	
High school	2.74 (1.01–7.45)		4.16 (2.6–6.65)	
Some college or above	1 (0.49–2.05)		5.83 (2.12–16.03)	

Model 1: Adjusted for age, sex, race-ethnicity, and education levels; Model 2: Adjusted for variables in Model 1 plus smoking status, drinking status, vigorous, and moderate physical activity; Model 3: Adjusted for variables in Model 2 plus a history of diabetes, hypercholesterolemia, cardiovascular disease, stroke, and tumors.

**Table 5:** Hazard ratios of hypertension combined with cognitive impairment on all-cause and CVD-specific mortality in various subgroups among participants, NHANES, 2011–2014.

		HR (95% CI)		
Characteristic	No hypertension & no cognitive impairment	Hypertension & no cognitive impairment	No hypertension & cognitive impairment	Hypertension & cognitive impairment
All-cause mortality				
Age, years				
60–69	1 (Reference)	19.99 (5.94–67.28)	12.71 (0.87–186.84)	46.22 (10.83–197.2)
70–80	1 (Reference)	1.33 (0.72–2.45)	4.54 (1.68–12.26)	4.21 (1.91–9.28)
*p* for interaction		0	0.28	0
Sex				
Male	1 (Reference)	3.36 (1.6–7.08)	8.25 (1.93–35.36)	5.71 (2.42–13.48)
Female	1 (Reference)	1.5 (0.55–4.05)	4.67 (1.32–16.53)	5.21 (1.43–18.98)
*p* for interaction		0.31	0.64	0.63
Race				
Other race	1 (Reference)	2.15 (1.14–4.04)	5.61 (1.5–20.93)	6.77 (2.98–15.37)
Non-hispanic white	1 (Reference)	3.02 (1.08–8.42)	7.34 (2.25–23.92)	4.96 (1.74–14.1)
*p* for interaction		0.53	0.66	0.84
Education status				
<High school	1 (Reference)	0.94 (0.32–2.77)	1.9 (0.51–7.08)	2.09 (0.55–7.89)
High school	1 (Reference)	5.26 (1.7–16.31)	13.91 (3.82–50.63)	17 (4.83–59.77)
Some college or above	1 (Reference)	1 (0.47–2.16)	5.85 (0.83–41.06)	5.85 (1.31–26.2)
*p* for interaction		0.67	0.68	0.32
CVD mortality				
Age, years				
60–69	1 (Reference)	2.37 (1.09–5.16)	4.4 (0.82–23.72)	5.15 (2.44–10.88)
70–80	1 (Reference)	1.23 (0.92–1.66)	2.12 (1.31–3.42)	2.25 (1.38–3.67)
*p* for interaction		0.18	0.38	0.04
Sex				
Male	1 (Reference)	1.62 (1.02–2.58)	2.61 (1.25–5.45)	2.49 (1.48–4.22)
Female	1 (Reference)	1.5 (1.01–2.24)	2.17 (1.02–4.65)	2.82 (1.56–5.08)
*p* for interaction		0.55	0.9	0.19
Race				
Other Race	1 (Reference)	1.97 (1.3–2.96)	2.98 (1.58–5.6)	2.56 (1.72–3.83)
Non-Hispanic White	1 (Reference)	1.48 (1.1–1.99)	2.75 (1.48–5.1)	3.17 (1.9–5.28)
*p* for interaction		0.27	0.12	0.52
Education status				
<High school	1 (Reference)	0.75 (0.45–1.26)	1.34 (0.71–2.53)	1.41 (0.77–2.59)
High school	1 (Reference)	2.36 (1.66–3.34)	4.61 (2.52–8.44)	5.23 (2.92–9.37)
Some college or above	1 (Reference)	1.15 (0.69–1.91)	0.7 (0.17–2.92)	2.36 (0.97–5.72)
*p* for interaction		0.88	0.85	0.17

Model 1: Adjusted for age, sex, race-ethnicity, and education levels; Model 2: Adjusted for variables in Model 1 plus smoking status, drinking status, vigorous, and moderate physical activity; Model 3: Adjusted for variables in Model 2 plus a history of diabetes, hypercholesterolemia, cardiovascular disease, stroke, and tumors.

A set of sensitivity analyses was conducted to validate the reliability of the primary findings in this study. The main results of this study remained robust after excluding participants who died within one year and those with a history of CVD, stroke, or cancer, as presented in Supplementary Tables S1–S8.

## Discussion

The present study recruited 2,925 participants, representing a weighted population of 53,086,905 Americans aged 60 years and older. The weighted prevalence of hypertension and CI in this population was 59.1% and 10.3%, respectively. During the follow-up period of up to 9.17 years, or 18,731.56 (weighted 3.46 × 10^8^) person-years, a total of 576 deaths occurred. The results indicated that individuals with hypertension or CI had significantly higher all-cause mortality and CVD-specific mortality compared to those without these conditions. Furthermore, the joint analysis demonstrated that participants with both hypertension and CI had a significantly increased risk of all-cause mortality and CVD-specific mortality when compared to individuals with either hypertension or CI alone. Notably, these findings showed stronger associations in the younger subgroup (aged 60–69 years) than in the older subgroup (aged 70–80 years) during the age-stratified analysis.

Several studies have demonstrated that individuals with hypertension have significantly higher mortality rates related to all causes and CVD compared to those without hypertension (19–21). A recent study indicated that these associations were more pronounced in individuals who developed hypertension before the age of 45, while the strength of these associations decreased with advancing age (22). Another study discovered a U-shaped relationship between hypertension and mortality in older adults, with the lowest risk observed at a blood pressure level of 160 mm Hg (95% CI: 154–181 mm Hg; *p* < 0.001) (23). Importantly, untreated white-coat hypertension was also found to be associated with an increased risk of cardiovascular events and mortality from all causes (24). In developing countries, the risk of mortality due to hypertension is greater compared to developed countries, and there is also a heavier burden associated with treatment. Results from the China Kadoorie Biobank Study revealed that approximately one-third of Chinese adults had hypertension, with significantly lower rates of diagnosis, treatment, and control compared to Western populations, consequently leading to excess mortality (25).This study further validates the associations between hypertension and mortality from all causes and CVD, and highlights the stronger links in younger patients compared to older patients, which is consistent with previous research findings.

The appropriateness of lowering systolic blood pressure in older adults remains controversial. Findings from studies on antihypertensive treatment suggested that antihypertensive therapy had a long-term beneficial effect on reducing mortality and cognitive function (26, 27). Conversely, results from the population-based Leiden 85-plus cohort study suggested that a lower systolic blood pressure in elderly individuals taking antihypertensive medication was associated with higher mortality and faster cognitive decline (28). According to a study from China, CI was not directly associated with hypertension in Chinese nonagenarians and centenarians (29). These results suggest that further exploration is needed to understand the effects of antihypertensive treatment in the elderly population.

Previous studies have demonstrated a strong association between CI and excess mortality. A cohort study revealed that both mild and moderate to severe CI were associated with an increased mortality hazard, independent of other mortality risk factors [HR, 1.184 (95% CI: 1.051–1.334) and for mild impairment; HR, 1.447 (95% CI: 1.235–1.695) for moderate to severe impairment] (30). The results of the Sydney Memory and Ageing Study indicated that both baseline cognitive ability and the decline in cognitive ability over a span of 2 years were predictive of mortality (31). Additionally, cognitive decline remained a significant predictor of mortality when considering other medical risk factors. Another study demonstrated a significant association between CI and 1-year mortality after major surgery in older adults (7). The results of the current study aligned with the previous findings. It is noteworthy that current statistical methods for assessing mortality have the potential to underestimate the impact of dementia-related mortality (6).

Several previous studies have demonstrated that individuals with hypertension have a higher susceptibility to CI when compared to those without hypertension, particularly when accompanied by diabetes mellitus, metabolic syndrome, and other comorbidities (8–10, 32). According to one study's findings, the association between hypertension and cognitive function varied by age, with a more pronounced correlation observed among individuals over the age of 60 (33). Furthermore, the risk of CI among individuals with hypertension was strongly associated with the age of onset of hypertension, blood pressure levels, as well as the presence of frailty and hyperglycemia (10, 34–36). Studies suggested that endothelial dysfunction was involved in both hypertension and cognitive impairment (10, 11). Several medications, such as L-arginine, empagliflozin, choline, metformin, and others, have been found to improve endothelial function and may therefore be beneficial in enhancing cognitive function (12, 37–39).

The relationship between hypertension and CI in the elderly population remains incompletely understood. A study carried out in Kangwha County, South Korea, reported that individuals with concurrent CI and hypertension exhibited a greater risk of all-cause mortality compared to those with either CI or hypertension alone (40). A study conducted in southern Italy demonstrated that cognitively impaired patients with the lowest and highest diastolic blood pressures experienced the highest relative risk of mortality (41). The findings of a study involving older adults aged 80 years or older indicated that individuals in the oldest age group experiencing an annual decline of more than one point in the MMSE score were associated with an approximately 4% risk of all-cause mortality (42). Notably, hypertension exerted a statistically significant influence on this association (*p* = 0.004). Cognitively impaired patients exhibited a U-shaped trend regarding mean arterial and systolic blood pressure, wherein the risk of mortality increased in the presence of both very low and very high blood pressure levels (43). The study's results demonstrated that patients with both CI and hypertension exhibited notably higher rates of all-cause mortality and CVD-specific mortality when compared to patients without concurrent hypertension. These findings are consistent with previous studies.

However, contrary findings suggested that increased mortality attributed to CI may not be associated with hypertension. A study conducted in a limited sample of memory clinics revealed no detectable association between diurnal blood pressure variations and elevated total mortality rates in the elderly population with CI (44). The results of the Milan Geriatrics 75+ Cohort Study indicated a negative association between higher SBP and mortality risk reduction in individuals with impaired cognitive function (13).

Frailty is a common geriatric syndrome among the elderly. The prevalence of frailty in the elderly population ranges from 16% to 26.7% (45–47). Frail older adults have higher rates of mortality and various diseases, especially cardiovascular diseases such as hypertension, diabetes, and heart failure. Results from the NHANES survey indicated that frailty (HR = 2.76, 95% CI = 2.33–3.27) and pre-frailty (HR = 1.38, 95% CI = 1.19–1.59) were significantly associated with all-cause mortality (45). The National Trends in Health and Aging Study showed that during the 6-year follow-up period, frail patients had significantly higher rates of death and cardiovascular outcomes than non-frail patients, including major adverse cardiovascular events (MACE) (HR = 1.77, 95% CI = 1.53–2.06) and death (HR = 2.70, 95% CI = 2.16–3.38) (47). Hypertension was considered the most important independent risk factor predicting mortality in frail patients (48). The Systolic Blood Pressure Intervention Trial (SPRINT) study demonstrated that frail patients benefited from intensive blood pressure control without an increased risk of serious adverse events (46). Therefore, efforts are needed to recognize frailty in the elderly population, and interventions to limit or reverse frailty may reduce the incidence of adverse cardiovascular events and prolong survival expectations in frail older adults.

The present study possessed multiple strengths. A key advantage was the utilization of a sample of older adults extracted from the NHANES database, a nationally representative dataset that improved the generalizability of our findings to U.S. adults aged ≥60. Furthermore, mortality risk was accurately assessed through the utilization of the National Death Index. Thirdly, we conducted a series of stratified and sensitivity analyses to exhibit the robustness and consistency of our study results.

The present study has several limitations. Firstly, the study had a relatively small sample size due to cognitive function tests being conducted only during a specific annual cycle (2011–2014) within the NHANES population. Moreover, a more extended follow-up period is necessary to further validate the relatively brief duration of the follow-up period. Thirdly, while the primary analysis considered potential confounding variables related to personal characteristics, lifestyle patterns, and chronic medical records at the baseline, there remains a possibility that residual and unmeasured confounders may have influenced the research findings. In addition, the following potential biases may have been present in this study. One possible source of bias was the selection bias that may have occurred during the recruitment process. The other possible source of bias was measurement bias. Many of the sociodemographic variables, lifestyles, and comorbidities of the participants in this study were provided by self-report or recall, and thus there may have been self-report bias or recall bias. In addition, although in the regression model we controlled for a few parametric variables that could affect the results, it is likely that there are other variables that we did not consider that could affect the results of this paper. Lastly, it is important to exercise caution when extrapolating the results of the present study, as they are based on a nationally representative sample of individuals from the United States and may not be fully applicable to distinct racial and ethnic groups beyond the United States.

## Conclusion

According to the findings of the current study, patients with CI experience significantly higher rates of both overall mortality and mortality related to CVD compared to than non-CI patients. Furthermore, elderly patients with CI who also have hypertension face an even greater risk of mortality. Appropriate management of hypertension in patients with CI may be helpful in reducing the excess risk of death.

## Data Availability

The original contributions presented in the study are included in the article/**Supplementary Material**, further inquiries can be directed to the corresponding authors.
