# Supplementary material for: Effect of blood pressure on mortality in patients with cognitive impairment: a prospective cohort study
Source: Front Cardiovasc Med. 2023 Dec 12;10:1282131. doi: 10.3389/fcvm.2023.1282131 (PMC10754517; doi:10.3389/fcvm.2023.1282131)
Supplement: Supplementary file 1 [file Presentation1.zip › Presentation 1_v1/supplementary materials/Supplementary materials.docx]

Supplementary materials

Table S1. Hazard ratios of Hypertension and Cognitive Impairment with All-cause and CAD-specific Mortality After Excluding Participants Who Died Within 1 Year

Table S2. Hazard ratios of Hypertension Combined with Cognitive Impairment on All-cause and CAD-specific Mortality After Excluding Participants Who Died Within 1 Year

Table S3. Hazard ratios of Hypertension and Cognitive Impairment with All-cause and CAD-specific Mortality After Excluding Participants with a History of CAD

Table S4. Hazard ratios of Hypertension Combined with Cognitive Impairment on All-cause and CAD-specific Mortality After Excluding Participants with a History of CAD

Table S5. Hazard ratios of Hypertension and Cognitive Impairment with All-cause and CAD-specific Mortality After Excluding Participants with a History of Cancer

Table S4. Hazard ratios of Hypertension Combined with Cognitive Impairment on All-cause and CAD-specific Mortality After Excluding Participants with a History of Cancer

Table S7. Hazard ratios of Hypertension and Cognitive Impairment with All-cause and CAD-specific Mortality After Excluding Participants with a History of Stroke

Table S8. Hazard ratios of Hypertension Combined with Cognitive Impairment on All-cause and CAD-specific Mortality After Excluding Participants with a History of Stroke
